# Identification of Cathepsin D as a Plasma Biomarker for Alzheimer’s Disease

**DOI:** 10.3390/cells10010138

**Published:** 2021-01-12

**Authors:** Jae-Whan Kim, Soon-Young Jung, Youngbin Kim, Hansol Heo, Chang-Hyung Hong, Sang-Won Seo, Seong-Hye Choi, Sang-Joon Son, Seongju Lee, Jaerak Chang

**Affiliations:** 1 Department of Biomedical Sciences, Ajou University School of Medicine, Suwon 16499, Korea; jaewhan55@ajou.ac.kr (J.-W.K.); syjjeong@ajou.ac.kr (S.-Y.J.); curlyoung@ajou.ac.kr (Y.K.); loper2@ajou.ac.kr (H.H.); 2 Program in Biomedical Science and Engineering, Department of Anatomy, College of Medicine, Inha University, Incheon 22212, Korea; 3 Department of Psychiatry, Ajou University School of Medicine, Suwon 16499, Korea; antiaging@ajou.ac.kr; 4 Samsung Medical Center, Department of Neurology, Sungkyunkwan University School of Medicine, Seoul 06351, Korea; sangwonseo@skku.edu; 5 Department of Neurology, Inha University School of Medicine, Incheon 22212, Korea; seonghye@inha.ac.kr; 6 Department of Brain Science, Ajou University School of Medicine, Suwon 16499, Korea

**Keywords:** cathepsin D, Alzheimer’s disease, plasma biomarker, CDR-SB score

## Abstract

Although Alzheimer’s disease (AD) is the most common neurodegenerative disease, there are still no drugs available to treat or prevent AD effectively. Here, we examined changes in levels of selected proteins implicated in the pathogenesis of AD using plasma samples of control subjects and patients with cognition impairment. To precisely categorize the disease, fifty-six participants were examined with clinical cognitive tests, amyloid positron emission tomography (PET) scan, and white matter hyperintensities scored by magnetic resonance imaging. Plasma cathepsin D levels of the subjects were examined by immunoblotting and enzyme-linked immunosorbent assay (ELISA). Correlation of plasma cathepsin D levels with AD-related factors and clinical characteristics were examined by statistical analysis. By analyzing quantitative immunoblot and ELISA, we found that the plasma level of cathepsin D, a major lysosomal protease, was decreased in the group with amyloid plaque deposition at the brain compared to the control group. The level of plasma cathepsin D was negatively correlated with clinical dementia rating scale sum of boxes (CDR-SB) scores. In addition, our integrated multivariable logistic regression model suggests the high performance of plasma cathepsin D level for discriminating AD from non-AD. These results suggest that the plasma cathepsin D level could be developed as a diagnostic biomarker candidate for AD.

## 1. Introduction

Dementia is a group of symptoms associated with a gradual decline in memory and thinking abilities. Since dementia occurs more frequently in the aged group, increased life expectancy also increases the prevalence of dementia, which will inevitably increase social costs. Various genetic and environmental factors are known to cause dementia. Alzheimer’s disease (AD) and vascular dementia are the most common types of dementia. AD is characterized by the accumulation of extracellular amyloid-beta (Aβ) plaques and intracellular neurofibrillary tau tangles in the brain, leading to synaptic dysfunction and neuronal loss [1,2]. Given that AD develops initially without obvious symptoms over several years, early diagnosis during the latent period is essential for treatment with a hopeful prognosis.

Currently, structural magnetic resonance imaging (MRI), amyloid positron emission tomography (PET) scans, and cerebrospinal fluid (CSF) analysis of Aβ or total tau protein (T-tau) and phosphorylated tau (p-tau) levels are considered reliable diagnostic methods for AD to aid clinical assessment [3,4]. However, these diagnostics are expensive and require invasive lumbar puncture. Thus, several efforts have been made to find blood-based biomarkers to develop more convenient, inexpensive, and non-invasive diagnostic ways for AD diagnosis. However, previous studies with established CSF AD biomarkers have shown conflicting results on their usefulness as plasma biomarkers for AD diagnosis [5,6,7]. For example, CSF Aβ42 levels were significantly decreased in AD patients, whereas serum or plasma Aβ42 levels in AD patients were not significantly different from those in a control group [8]. Nevertheless, its convenience, simplicity, and cheapness lead many researchers to make efforts to develop and find novel reliable plasma biomarkers for AD.

Cathepsin D encoded by the *CTSD* gene is a lysosomal aspartic acidic protease. It exists as a diglycosylated precursor form (pro-cathepsin D) in the Golgi complex. Once pro-cathepsin D loses its inhibitory signal peptide, it becomes a mature and active enzymatic form through further processing in the lysosome [9,10,11]. Lysosomal cathepsin D mediates degradation of unfolded protein aggregates delivered from endocytosis or autophagy. The autophagy-lysosome pathway is thought to be the main clearance system for Aβ and tau [12]. Cathepsin D is also implicated in the pathogenesis of AD due to its involvement in the processing of APP and tau [11,13,14,15]. Furthermore, cathepsin D is highly expressed in cortical and hippocampal neurons of the postmortem brains of AD patients [16]. Cathepsin D levels are abnormally elevated in amyloid plaques of brains and CSF of AD patients [17,18,19,20]. Although these studies strongly suggest that cathepsin D could be a reliable biomarker for AD, there are still inconsistent results after examining cathepsin D levels in blood samples of AD patients. Consistent with previous studies, increased levels of cathepsin D have been reported from a study using plasma samples of AD patients [21]. On the contrary, another study has reported the down-regulation of cathepsin D levels in fibroblasts derived from AD patients [22]. Decreased expression of cathepsin D was also observed in monocytes of AD patients [23]. Therefore, further study is needed to resolve these conflicting results on cathepsin D levels in the plasma of AD patients to determine whether cathepsin D could be used as a plasma biomarker for AD.

In this study, we aimed to determine whether plasma cathepsin D could be a useful biomarker for AD diagnosis. We found that plasma cathepsin D level was significantly decreased in the AD group and positively correlated with cognitive standards. Furthermore, our biomarker model, including co-variates and cathepsin D, showed high performance in distinguishing AD from the non-AD group. Our results suggest that the cathepsin D level in plasma could be potentially developed as a biomarker for the diagnosis and prediction of AD.

## 2. Materials and Methods

### 2.1. Subjects and Study Approval

Participants were recruited by Biobank Innovations for chronic Cerebrovascular disease With Alzheimer’s disease Study (BICWALZS) and the Center for Convergence Research of Neurological Disorders. Of them, we analyzed 56 patients with available amyloid positron emission tomography (PET), 3D T1 MRI data, and blood samples. The clinical diagnostic criteria used for this study are as follows: The control subjects had no cognitive impairment with normal amyloid PET results and a white matter hyperintensity (WMH) score of 1. Mild cognitive impairment (MCI) was evaluated based on a Clinical Dementia Rating (CDR) [24] score of 0.5 under the expanded Mayo Clinic criteria [25]. The patients had mild cognitive impairment with normal amyloid PET results and a WHM score of 1. AD dementia was evaluated under the National Institute on Aging-Alzheimer’s Association core clinical probable AD dementia criteria [26]. The patients had cognitive impairment with abnormal amyloid PET findings and WMH score of 1. Vascular dementia (VaD) was evaluated under the Diagnostic Statistical Manual of Mental Disorders, fifth edition. The patients had cognitive impairment with normal amyloid PET results and WMH score of 2 or 3. We excluded patients with at least one of the following criteria: (1) possible behavioral variant frontotemporal lobar degeneration; (2) possible Lewy body dementia; and (3) history of neurological or medical conditions such as territorial cerebral infarction, intracranial hemorrhage, Parkinson’s disease, heart failure, renal disease. Written informed consent was obtained from all participants and caregivers. This study was approved by the Ajou Institutional Review Board (AJIRB-BMR-KSP-17-0376).

### 2.2. Plasma Sample Preparation and Immunoblotting

Albumin and IgG were removed from the plasma by using Hi-Bind Albumin-IgG depletion beads (Biovision, Milpitas, CA, USA), following the manufacturer’s instructions. Albumin- and IgG-depleted plasma samples were mixed with 2X SDS-loading buffer and then boiled at 95 °C for 10 min. Immunoblotting was performed as previously described [27]. Briefly, samples were separated by SDS-PAGE and transferred onto a nitrocellulose membrane. The membrane was blocked with 3% skim milk in Tris-buffered saline containing 0.05% Tween-20 (TBST). Blots were incubated with primary antibodies diluted in TBST with 3% BSA, washed with TBST, and incubated with secondary antibodies conjugated with horseradish peroxidase in 3% skim milk in TBST. Primary antibodies specific to cathepsin D (Calbiochem, San Diego, CA, USA, IM-03, mouse mAb, clone BC011) and α-Tubulin (Abcam, Cambridge, MA, USA, ab18251, rabbit pAb, derived from residues 400 to the C-terminus of human α-Tubulin) were purchased. Immunoreactive proteins were detected with a ChemiDoc Gel Imaging System (Bio-rad). Band intensities were measured using Image Lab software (Bio-rad, Hercules, CA, USA).

### 2.3. Antibody Validation

For the gene editing of cathepsin D, two targets (CR1: TCCAGGTGGACCTGCCAGTAGG; CR2: GCCTACTGGCAGGTCCACCTGG) were selected from the list recommended by E-CRISP program (http://www.e-crisp.org/E-CRISP). After cloning of gRNAs specific to the targets into pSpCas9 (BB)-2A-Puro (PX459) vector (Addgene, #48139), the constructs were transfected into HeLa cells with Avalanche-Omni reagents (EZ Biosystems, College Park, MD, USA) for 36 h, followed by incubation with 2 μg/mL of puromycin for 24 h. After further growth, the cells were lysed and boiled with 1X SDS lysis buffer and subjected to immunoblotting.

### 2.4. ELISA

An enzyme-linked immunosorbent assay (ELISA) kit for cathepsin D (Wuhan USCN, Wuhan, China, SEB280Hu) was purchased, and the assay was performed according to the manufacturer’s instructions. Briefly, plasma samples were diluted to one-fifth with Dulbecco’s phosphate-buffered saline (DPBS), and standard solutions were prepared by serial dilution. Samples and standards were incubated with antibody-coated wells for 1 h. After discarding the solutions, the wells were incubated with detection reagent A for 1 h, washed three times, incubated with detection reagent B for 30 min, and washed five times. Finally, the substrate solution was applied to each well until the blue color sufficiently appeared. The reaction was then stopped by adding stop solution, and the plate was read immediately at a wavelength of 450 nm using a plate reader (Synergy H1, BioTek, Winooski, VT, USA).

### 2.5. APOE Genotyping

Informed consent about collecting and genotyping blood genomic DNA samples was obtained from all participants. Genomic DNA was isolated from the blood samples, and SNP (single-nucleotide polymorphism) genotyping was performed by DNA Link (Seoul, Korea) using Affymetrix Axiom KORV1.0-96 Array (Thermo Fisher Scientific, Waltham, MA, USA) according to the manufacturer’s protocol. *APOE* genotypes were derived from rs429358 and rs7412, which were included in the array.

### 2.6. Statistical Analysis

Results were expressed as mean ± SD (standard deviations). Logistic regression analysis was performed to integrate the plasma cathepsin D level with age, sex, and presence of an apolipoprotein ε4 allele. Receiver operating characteristic (ROC) curve analysis and Pearson’s chi-square test were performed to assess the accuracy of biomarker models. GraphPad Prism 7 was used to analyze statistical data and draw graphs. Significance levels for comparisons among groups were determined by Kruskal–Wallis test with Dunn’s multiple comparisons post-hoc test. Significance between the two groups was determined by a Mann–Whitney U test. *p* < 0.05 was considered significant.

## 3. Results

### 3.1. Preparation of Patient Plasma Samples for SDS-PAGE Analysis

To establish a convenient and easy assay system for AD diagnosis, we evaluated plasma cathepsin D levels using immunoblotting and ELISA. Since cathepsin D exists in various precursors and mature forms, we decided to use immunoblotting assay, which is suitable for distinguishing different-sized proteins in precursor and mature forms [11]. Compared to the analysis of intracellular proteins, interpretation of immunoblot results with plasma samples should be made carefully because the plasma is composed of highly complex components, and the immunoblot-based identification of plasma proteins has not been fully established.

**Figure 1:**
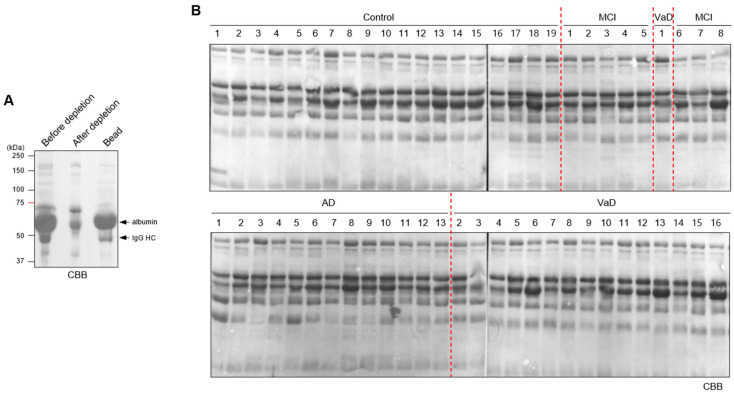
Preparation of patient plasma samples for SDS-PAGE analysis. (**A**) Plasma samples for SDS-PAGE analysis were prepared by depletion of affinity-purified albumin and immunoglobulin G (IgG) from the plasma. (**B**) Preparation of albumin/IgG-depleted control and patient plasma samples was verified by SDS-PAGE. CBB, Coomassie Brilliant Blue. HC, heavy chain. AD, Alzheimer’s disease; MCI, mild cognitive impairment; VaD, vascular dementia. Molecular weight standards (in kDa) are shown to the left.

Prior to quantitative analysis of plasma cathepsin D, albumin and immunoglobulin, the major components of plasma, were depleted because they could interfere with the resolving of samples on the polyacrylamide gel and the detection of specific bands by immunoblotting [28] (Figure 1A). The albumin/IgG-depleted plasma samples of control subjects and patients grouped into Control (*n* = 19), MCI (*n* = 8), AD (*n* = 13), and VaD (*n* = 16) were successfully resolved in the gel (Figure 1B; Table 1).

### 3.2. The Level of Cathepsin D Decreases in the AD Patient Plasma

**Figure 2:**
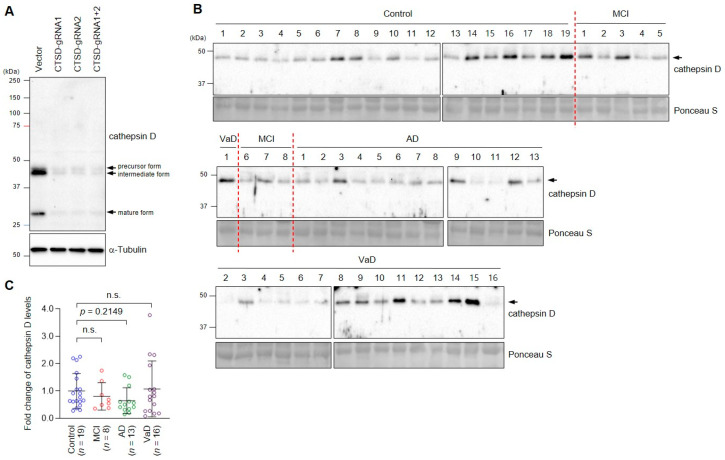
Immunoblotting analysis revealed that the level of cathepsin D decreased in the AD patient plasma. (**A**) Validation of the cathepsin D antibody for immunoblot analysis. HeLa cells were transfected with an empty vector (Vector) or vectors with gRNAs specific to the *CTSD* gene (CTSD-gRNA1, CTSD-gRNA2, CTSD-gRNA1+2), and lysates were immunoblotted with the indicated antibodies. (**B**) Analysis of the cathepsin D level of plasma samples from the control group and each patient group. Albumin/IgG-depleted plasma samples were immunoblotted with a cathepsin D antibody. Ponceau S staining was performed after SDS-PAGE to show that equal amounts of plasma proteins were loaded in each lane. (**C**) The band intensities of cathepsin D (B, arrow) in each patient group were compared to those of the control group. Each colored dot indicates the cathepsin D level of plasma samples from each patient, which was analyzed by immunoblotting. Mean ± SD was illustrated as a horizontal line in each group. The *p*-value was calculated using the Kruskal–Wallis test, followed by Dunn’s multiple comparisons test. n.s., not significant. Arrows indicate specific bands of cathepsin D. Molecular weight standards (in kDa) are shown to the left.

First, to validate the specificity of the cathepsin D antibody used in this study, we utilized a CRISPR/Cas9-mediated knockout system. Lysates from control cells and cathepsin D-specific gRNA-transfected cells were analyzed by immunoblotting for cathepsin D. As previously reported [11], three bands corresponding to precursor, intermediate, and mature forms were detected by the cathepsin D antibody in control cells (Figure 2A). All bands were faintly detected in cathepsin D-depleted cells, indicating that the antibody could specifically detect cathepsin D at endogenous levels.

Next, we examined plasma cathepsin D levels of control subjects and patients. In human plasma samples, the cathepsin D antibody detected one major band at 48 kDa, which corresponds to the intermediate form of cathepsin D (Figure 2B). Although the samples were loaded in separate gels due to the limited number of lanes, all SDS-PAGE and immunoblot analyses were performed at the same time under the same condition, including exposure time. Given that the band corresponding to the mature form of cathepsin D was faintly detected only in some samples (not shown), we concluded that the 48 kDa-band detected by the cathepsin D antibody was the major processed form of cathepsin D that was present in plasma. Next, we measured the intensity of the band to compare plasma cathepsin D protein levels between the groups. The MCI and VaD groups barely showed changes in plasma cathepsin D levels (0.80 ± 0.50, 1.07 ± 0.99, respectively) compared to the control (1.00 ± 0.64) (Figure 2C). On the other hand, although not statistically significant (*p* = 0.2149), relative plasma cathepsin D levels showed a tendency to decrease in the AD group (0.65 ± 0.48) (Figure 2C).

### 3.3. The Level of Plasma Cathepsin D Does Not Correlate with Clinical Characteristics Potentially Related to AD

**Figure 3:**
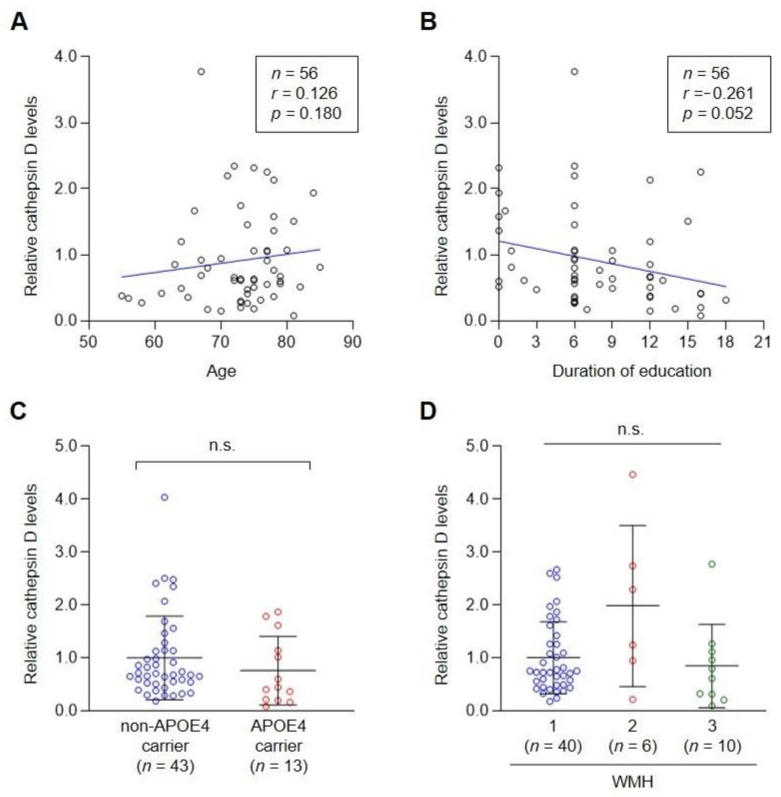
The level of plasma cathepsin D did not correlate with age, duration of education, the presence of the APOE ε4 allele, or WMH scores. (**A**–**D**) Correlations of plasma cathepsin D levels with age (**A**), duration of education (**B**), presence of the *APOE4* gene (**C**), and WMH scores (**D**) were analyzed. Each dot indicates the cathepsin D level of each control or patient plasma. (**A**,**B**) Pearson correlation coefficient (r) and *p*-value of linear regression are shown in each panel. (**C**,**D**) Mean ± SD was illustrated as a horizontal line in each group. n.s., not significant (Mann–Whitney U test for (**C**) or Kruskal–Willis test for (**D**)).

Next, we investigated the relationship between plasma cathepsin D levels and clinical characteristics potentially related to AD. Age is highly correlated with late-onset AD. A previous study revealed that serum cathepsin D levels were correlatively reduced in elderly healthy people [29]. However, plasma cathepsin D levels had no specific correlation with age in our study of mixed groups (Figure 3A). In addition, we did not find any correlation with the duration of education of the subjects (Figure 3B).

The ε4 allele of apolipoprotein E (*APOE*) is the most well-established genetic risk factor for AD across many studies [30,31,32,33]. Approximately 80% of familial AD and 64% of sporadic AD late-onset cases have at least one APOE4 [31,33,34]. We found the level of plasma cathepsin D of APOE4 carriers (0.76 ± 0.65) was lower than that of non-APOE4 carriers (Figure 3C). Although the difference was not statistically significant between the two groups, our results showing the decrease of plasma cathepsin D levels were consistent with a previous report demonstrating decreased cathepsin D levels in fibroblasts of AD patients [22]. We also examined the relationship between plasma cathepsin D levels and white matter hyperintensity (WMH) scores, which were used as a deciding factor to group VaD in this study. Although plasma cathepsin D levels seemed to increase in the group with WMH = 2 (1.98 ± 1.57), the increase was not significant among the groups.

### 3.4. The Level of Plasma Cathepsin D Negatively Correlates with CDR-SB Scores

It has been recently suggested that the diagnosis of AD is more appropriate if it is made by A/T/N (amyloid/tau/neurodegeneration) classification system, which is more objective and quantitative than conventional AD diagnosis methods [35]. However, since cognitive impairment, a typical symptom in patients with dementia, has been widely used as a standard for AD diagnosis, we examined the correlation between cognitive abilities and the level of plasma cathepsin D.

**Figure 4:**
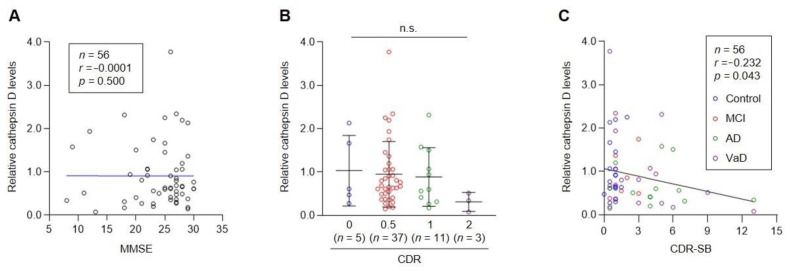
The level of plasma cathepsin D negatively correlates with CDR-SB scores. (**A**–**C**) Correlations of plasma cathepsin D levels with MMSE scores (**A**), CDR scores (**B**), and CDR-SB scores (**C**) were analyzed. Each dot indicates the cathepsin D level of each control or patient plasma. (**A**,**C**) Pearson correlation coefficient (r) and *p*-value of linear regression are shown in each panel. (**B**) Mean ± SD is illustrated as a horizontal line in each group. n.s., not significant (Kruskal–Wallis test).

Our result showed that there was no correlation between Mini-Mental State Examination (MMSE) scores and the level of plasma cathepsin D (Figure 4A). Although the mean level of cathepsin D tended to decrease with increasing CDR (Clinical Dementia Rating) scores, the decrease was not statistically significant due to the limited number of subjects in each group (*p* = 0.2696, Figure 4B). While CDR is a composite score ranging from 0 to 3 based on the assessment of cognitive and functional performance in six areas, CDR-SB (CDR Sum of Boxes) is a total score of all six areas. Therefore, CDR-SB is used as a tool to accurately stage the severity of dementia [5]. Intriguingly, the level of plasma cathepsin D was negatively correlated with the CDR-SB score (Figure 4C, *r* = −0.232, *p* = 0.043), even though the correlation was not significant in each group, which might be due to the low number of subjects. These results indicate that the level of cathepsin D is partially associated with cognitive abilities, which is a major symptom of dementia.

### 3.5. The Reduction of Plasma Cathepsin D of AD Patients Was Confirmed by ELISA

**Figure 5:**
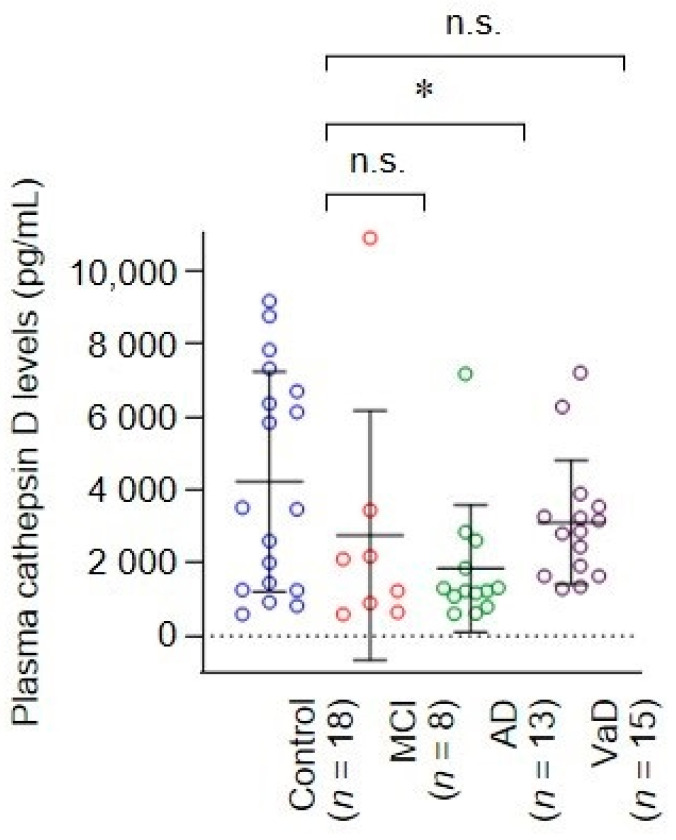
Enzyme-linked immunosorbent assay (ELISA) analysis revealed that the level of cathepsin D. * *p* < 0.05, n.s., not significant, Kruskal-Wallis test, followed by Dunn’s multiple comparisons test.

To confirm the decreased level of plasma cathepsin D in the AD group, we measured plasma cathepsin D levels by ELISA, which is a more common and convenient method for diagnosis than immunoblotting. ELISA results showed that the AD group had significantly lower plasma cathepsin D levels (1836 ± 1744 pg/mL) than controls (4219 ± 3011 pg/mL) (*p* = 0.0266, Dunn’s multiple comparisons test, Figure 5). MCI and VaD groups also had lower cathepsin D levels (2748 ± 3427 pg/mL and 3098 ± 1698 pg/mL, respectively) than the control group, but those were not statistically significant (Figure 5). Taken together, we revealed that the level of cathepsin D in AD patient plasma is significantly lower than that in control subjects based on the results of immunoblotting and ELISA. In addition, we found that the level of plasma cathepsin D is also associated with decreased cognitive abilities. 

### 3.6. Multivariate ROC Curve Analysis Validated Plasma Cathepsin D as a Biomarker for AD

Finally, we evaluated the performance of plasma cathepsin D levels using a statistical prediction model, ROC curve analysis. The ROC curve represents the sensitivity as a function of the specificity, and the area under the curve (AUC) indicates the accuracy of a model to discriminate between the two groups. 

**Figure 6:**
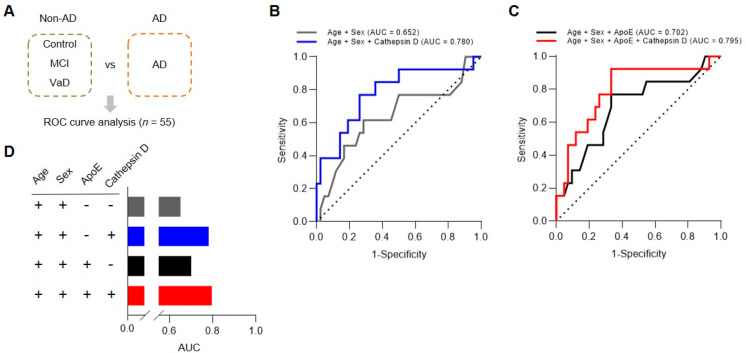
Analysis of plasma cathepsin D biomarker performance in distinguishing AD from non-AD subjects. (**A**) Categorized subject groups for logistic regression analysis. (**B**–**D**) Comparison of ROC curve among combinations of variables (age, sex, ApoE, cathepsin D). Details are described in Table 2.

We performed logistic regression analysis, followed by ROC curve analysis, to assess the performance of plasma cathepsin D levels for diagnosis of AD. Firstly, we re-grouped our study subjects into two groups, (1) Non-AD group including control, MCI, and VaD; (2) AD group, for multivariate modeling (Figure 6A). Using two groups, first, we plotted a ROC curve and calculated AUC with co-variates, such as age and sex (Figure 6B,D; AUC = 0.652). However, the AUC was higher with plasma cathepsin D levels from the ELISA results to the ROC curve analysis (Figure 6B,D; AUC = 0.780). The AUC was also increased from 0.652 to 0.702 when the genetic information on the presence of an APOE4 allele was included and further increased by including plasma cathepsin D levels (Figure 6C,D; AUC = 0.795). Thus, the performance of our biomarker models was increased by which cathepsin D had a positive effect on AUC with good diagnostic accuracy based on AUC interpretation criteria (0.9–1.0 = excellent, 0.8–0.9 = very good, 0.7–0.8 = good, 0.6–0.7 = sufficient, 0.5–0.6 = bad, <0.5 = not useful) [36,37].Taken together, our findings suggest that plasma cathepsin D could be developed as a potential biomarker for the diagnosis of AD.

## 4. Discussion

Despite numerous efforts to explore plasma biomarkers for AD for more than a decade, there is still no remarkable results comparable to CSF biomarkers [6,7]. In this research, we sought to examine the possibility of applying cathepsin D as a plasma biomarker for AD diagnosis. By immunoblotting and ELISA, we found that the level of plasma cathepsin D was decreased in patients with amyloid defects (AD group). We also found that the level of plasma cathepsin D negatively correlates with the CDR-SB score, which is one of the commonly used clinical cognitive standards for AD diagnosis. The above results suggest that the level of plasma cathepsin D could be a novel biomarker for AD diagnosis.

A molecular biomarker is defined as a molecular characteristic that indicates normal physiology and pathological process. Thus, it is useful not only for the diagnosis of diseases but also for the development and design of new drugs. A lysosomal hydrolase, cathepsin D, is an attractive candidate for AD biomarker. Alteration of cathepsin D expression has been often reported in studies using blood and CSF samples of AD patients [38]. Moreover, cathepsin D is clearly associated with the pathogenesis of AD because it is directly implicated in the degradation of Aβ and processing of tau [39,40]. The association between genetic variations of cathepsin D and neurodegenerative diseases has been reported [41,42]. Several missense mutations in CTSD were involved in brain atrophy and visual and motor problems, which are associated with several neurodegenerative diseases [43]. Recently, a study has reported that genetic deletion of cathepsin D induced the accumulation of cerebral Aβ42, by which differentially degraded Aβ42 and Aβ40 [44]. In addition, lysosomal exo- and endo-proteases including cathepsin D can regulate neuronal cell apoptosis, by which decreased lysosomal proteases leads to defects in the final step of autophagy that is important for neuronal cell survival [45]. It has been reported that cathepsin-mediated apoptosis is accompanied by antiapoptotic Bcl-2 family protein cleavage by cytosolic cathepsins [46]. Another study showed that the localization and balance of cathepsins are related to AD [47]. These studies indicate lysosomal proteases are tightly associated with the survival of neuronal cells. 

The association of cathepsins with various diseases other than neurodegenerative diseases have also been thoroughly studied [48,49]. In proliferative diabetic retinopathy, decreased cathepsins were found in the vitreous humor, serum, and blood cells. The expression of cathepsin B, cathepsin D, and cathepsin L in retinal vascular endothelial cells decreased after treatment with high glucose, which induces apoptosis of retinal vascular endothelial cells [50]. It has been reported that plasma pro-cathepsin D levels were elevated in breast cancer patients [51]. As pro-cathepsin D promotes cell proliferation, elevated pro-cathepsin might be beneficial to cancer cell growth. A study showed that upregulation of cathepsin D protected against cardiac malfunction by which promoted myocardial autophagic flux in an ischemic heart disease model [52].

Nevertheless, there have been limited trials to examine the possibility of cathepsin D as an AD biomarker, and even the results were inconsistent [21]. Except for a study that analyzed levels of lysosomal enzymes in CSF samples by immunoblotting, ELISA assays were performed to assess cathepsin D levels in most studies [53]. We speculated that the inconsistency of previous studies might be due to the existence of multiple forms of cathepsin D. Therefore, we performed immunoblotting, which allows separation of different forms of cathepsin D. As a result, we could detect a single major cathepsin D form that was decreased in the AD group. The decrease was consistently observed in ELISA. Our results support previous studies showing a decrease in cathepsin D levels in AD patients [22,23]. Importantly, the statistical model, including plasma cathepsin D levels analyzed by logistic regression, showed high performance in distinguishing AD from non-AD (Table 2).

Although our biomarker model based on the level of plasma cathepsin D showed high performance in distinguishing AD patients from the non-AD group, there were two limitations of our study. First, the cathepsin D decrease in AD patient plasma should be further validated with a large number of patients. Second, more technically precise studies are necessary to determine which form of cathepsin D is decreased in the AD patient plasma. By biosynthetic processing, cathepsin D can exist in various forms [54]. Especially in our study, it was difficult to distinguish between intermediate cathepsin D and pseudo-cathepsin D with a similar molecular weight.

Earlier studies have shown that the level of cathepsin D is elevated in brain tissues and CSF of AD patients, suggesting that an increase in cathepsin D levels could be used as an AD biomarker [17,18,19,20,33,55]. However, more recent studies using fibroblasts and blood samples of patients have reported a decrease of cathepsin D levels in AD patients, consistent with our study [22,23]. Nonetheless, additional careful examinations are necessary to verify the relation of AD and the level of cathepsin D in plasma, given that peripheral cathepsin D levels could be different from those in the brain. Moreover, considering the implication of plasma components such as cholesterol, thrombin, and cytokines in AD [56,57,58,59], further correlation analysis of plasma cathepsin D with AD-related factors could help improve the performance of our AD biomarker model.

In conclusion, we found that the plasma cathepsin D level was significantly decreased in AD subjects, and it was also correlated with the cognitive abilities of the patients. Importantly, we suggest the possibility of applying plasma cathepsin D as a peripheral biomarker of AD, based on the high AUC value in our prediction model. It is still unclear whether the alteration in plasma cathepsin D levels is a cause or a result of AD. We hope that our study will lead to further research that investigates the role of peripheral cathepsin D in AD pathogenesis.

## Figures and Tables

**Table 1:** Demographic and clinical characteristics of subjects.

	Control (*n* = 19)	MCI (*n* = 8)	AD (*n* = 13)	VaD (*n* = 16)
Sex (male/female)	6/13	2/6	7/6	5/11
Age (years)	74 ± 3.4	67.8 ± 7.8	72.2 ± 7.6	74.7 ± 7.3
Education duration (years)	6.3 ± 3.5	6.0 ± 6.0	13.1 ± 3.7	6.6 ± 4.9
MMSE	26.9 ± 2.0	26 ± 3.0	24.4 ± 4.3	22.4 ± 5.2
CDR	0.3 ± 0.2	0.5 ± 0.0	0.6 ± 0.2	0.8 ± 0.5
CDR-SB	0.8 ± 0.4	1.7 ± 1.2	3.3 ± 2.6	3.7 ± 3.9
Amyloid PET	normal	normal	abnormal	normal
WMH	-	1	1	2 or 3

MMSE, Mini-Mental State Examination; CDR, Clinical Dementia Rating; CDR-SB, CDR Sum of Boxes; WMH, White Matter Hyperintensities.

**Table 2:** Detailed information on receiver operating characteristic (ROC) curve analysis.

AD vs. Non-AD	AUC	Sensitivity (%)	Specificity (%)	*p*-Value ^a^	Cut Off	95% CI of AUC
Age + sex	0.652	46.15	76.19	0.173	0.359	0.491 to 0.813
Age + sex + cathepsin D	0.780	61.54	80.95	0.036	0.278	0.625 to 0.909
Age + sex + ApoE	0.702	46.15	80.95	0.099	0.315	0.551 to 0.852
Age + sex + ApoE + cathepsin D	0.795	69.23	76.19	0.044	0.316	0.671 to 0.918

AUC, Area under the curve; CI, confidence interval, *n* = 55, ^a^ *p*-value by Pearson’s chi-square test.

## Data Availability

The data presented in this study are available on request from the corresponding author. The data are not publicly available due to ethical issue.
